# White Matter Microstructure Breakdown in the Motor Neuron Disease Spectrum: Recent Advances Using Diffusion Magnetic Resonance Imaging

**DOI:** 10.3389/fneur.2019.00193

**Published:** 2019-03-05

**Authors:** Silvia Basaia, Massimo Filippi, Edoardo G. Spinelli, Federica Agosta

**Affiliations:** ^1^Neuroimaging Research Unit, Institute of Experimental Neurology, Division of Neuroscience, Vita-Salute San Raffaele University, Milan, Italy; ^2^Department of Neurology, Institute of Experimental Neurology, Division of Neuroscience, San Raffaele Scientific Institute, Vita-Salute San Raffaele University, Milan, Italy

**Keywords:** amyotrophic lateral sclerosis, motor neuron disease, diffusion tensor imaging, fractional anisotropy, network analysis, magnetic resonance imaging, structural connectomics

## Abstract

Motor neuron disease (MND) is a fatal progressive neurodegenerative disorder characterized by the breakdown of the motor system. The clinical spectrum of MND encompasses different phenotypes classified according to the relative involvement of the upper or lower motor neurons (LMN) and the presence of genetic or cognitive alterations, with clear prognostic implications. However, the pathophysiological differences of these phenotypes remain largely unknown. Recently, magnetic resonance imaging (MRI) has been recognized as a helpful *in-vivo* MND biomarker. An increasing number of studies is applying advanced neuroimaging techniques in order to elucidate the pathophysiological processes and to identify quantitative outcomes to be used in clinical trials. Diffusion tensor imaging (DTI) is a non-invasive method to detect white matter alterations involving the upper motor neuron and extra-motor white matter tracts. According to this background, the aim of this review is to highlight the key role of MRI and especially DTI, summarizing cross-sectional and longitudinal results of different approaches applied in MND. Current literature suggests that DTI is a promising tool in order to define anatomical “signatures” of the different phenotypes of MND and to track *in vivo* the progressive spread of pathological proteins aggregates.

## Introduction

Motor neuron disease (MND) is a group of fatal neurodegenerative diseases characterized by progressive damage of the upper motor neurons (UMN) in the cortex and/or lower motor neurons (LMN) in the brainstem and spinal cord. Depending on the relative involvement of UMN and LMN, MND can be classified in a wide range of clinical phenotypes (including amyotrophic lateral sclerosis [ALS], primary lateral sclerosis [PLS], and progressive muscular atrophy [PMA]), characterized by different clinical presentation and progression rate. Advanced brain imaging techniques, such as magnetic resonance imaging (MRI), have been developed over the last decades in order to detect *in vivo* structural and functional brain abnormalities and to monitor neurodegeneration in the central nervous system of MND patients. Although neurodegeneration primarily affects the gray matter (GM), pathological alterations in the white matter (WM) have also been reported (1), involving not only the corticospinal tract (CST), but also non-motor regions (2).

The present review aims to discuss the current state of the art of MRI within different phenotypes of MND, focusing on WM microstructural alterations, underlining the role of MRI as a tool to understand disease pathophysiology and to provide potential biomarkers for diagnosis and prognostic stratification. Moreover, we also highlight emerging techniques, such as graph analysis, that will likely provide further insights in disease pathogenesis and might help in monitoring disease progression.

## Diffusion Tensor Imaging

### Basic Principles

Diffusion tensor imaging (DTI) is the most common MRI technique that allows to investigate WM microstructural changes. DTI is based on the random diffusion of water molecules in the fiber bundles, also known as Brownian motion (3). DTI analysis relies on the concept that, in a spherical volume, the diffusion of water has no preferential direction and spreads equally in three different directions (λ1, λ2, and λ3). Nevertheless, the movement of water molecules within the WM is approximately elliptical, having the greatest movement along axons (axial diffusivity [λ1]) caused by the restriction in the minor axes (radial diffusivity [λ2 and λ3]) imposed by myelin. In order to analyze the diffusion of water molecules, it is possible to define four parameters: (1) fractional anisotropy (FA), which describes how strongly directional is the movement of water molecules within the tissue; (2) radial diffusivity (RD, which is the average of λ2 and λ3); (3) axial diffusivity (AD, or λ1); (4) mean diffusivity (MD, obtained by the average of diffusion in the λ1, λ2, and λ3 axes). While the first three parameters (FA, RD, and AD) describe the spatial variation of water movement, MD reflects the average displacement of water molecules within the volume of interest. Axonal integrity will preserve diffusion parallel to the main fiber direction, resulting in higher FA and lower MD, while damage to the WM will lead to lower FA and higher MD (4). To date, there are several approaches to analyze DTI metrics: regions of interest (ROI) approach, whole-brain voxel-wise methods or tract-based spatial statistics (TBSS). These techniques provide complementary information and are characterized by relative strengths and limitations. The ROI approach is based on the delineation of defined areas or the reconstruction of WM tracts of interest in each subject's native space, in order to extract average DTI metrics to be compared among subjects; although this procedure allows a precise anatomical definition of WM structures and does not involve the coregistration of multiple scan images, it masks local alterations by averaging all voxels within the ROI, usually needs an *a priori* hypothesis and might be influenced by inter-subject anatomical variability (5). The most straightforward approach to assess local DTI alterations is to coregister all subjects' scans and perform statistical tests among groups within each voxel of the whole-brain WM mask; however, whole-brain voxel-wise approaches are sensitive to registration errors (6). To reduce the effects of local misregistrations, TBSS projects all voxels of the DTI image onto the nearest location on a “skeleton” delineating the main WM tracts (7). In addition to these methods, graph theory is one of the most recent approaches to investigate WM changes, building models of structural connectivity in brain disorders based on nodes and edges (8). Current evidence provided by each of these techniques for the study of MND is summarized in the following paragraphs.

The weakness of DTI is the lack of specificity in voxels presenting multiple fiber populations (termed “crossing fibers”) (9). In order to overcome this problem, novel data acquisition approaches have been proposed such as high angular resolution diffusion imaging (HARDI), neurite orientation dispersion and density imaging (NODDI) and diffusion spectrum imaging. Although these approaches hold the promise to provide further insights on the pathogenic mechanisms underlying WM degeneration and are likely sensitive to even subtle alterations in several neurodegenerative conditions (10), current evidence in the context of MND is scarce and should be considered preliminary (11, 12).

### DTI Signatures in ALS

Several studies have consistently demonstrated decreased FA and increased MD, RD, and AD along the entire CST in ALS patients relative to healthy controls (13–18). Several studies showed specific alterations of DTI metrics only in some parts of the CST: subcortical WM of the precentral gyrus, corona radiata, posterior limb of the internal capsule, cerebral peduncles and pons (19–21). DTI studies have also detected altered metrics in the middle and posterior part of the corpus callosum in ALS patients relative to healthy controls (22, 23). Cervical cord studies also consistently showed DTI alterations in the lateral columns of ALS patients (24–27), which were more severe at more distal cervical segments (25).

Many neuroimaging studies characterized the structural “signatures” in ALS patients with specific underlying genetic mutations. In particular, diffuse WM abnormalities were observed in *C9orf72* repeat expansion carriers (the most common genetic mutation) (28, 29). Particularly, *C9orf72* patients showed an involvement of the CST, whole corpus callosum and superior longitudinal fasciculus compared with healthy controls, in terms of decreased FA and increased MD (29). Only few structural MRI studies were performed in carriers of pathogenic mutations in *SOD1*, showing a relative preservation of brain motor networks compared to sporadic ALS patients (30, 31).

Cross-sectional DTI studies shed light on the pathophysiological processes associated with the development of ALS. However, the definition of biomarkers that could track progressive changes over time has crucial importance. To date, relatively few longitudinal studies focused on DTI changes over time in these patients, due to the difficulties in enrolling enough cases with a rapidly evolving disease who could undergo an appropriate number of follow-up scans. Most of the studies, using a ROI approach or TBSS, showed decreasing values of FA over time in CST, corpus callosum, frontal areas and cerebellum (21, 32–35). One study demonstrated also that diffusivity increased both in the external and internal capsule (21). Nevertheless, there are also studies showing inconsistent results, probably due to different sample sizes, follow-up intervals and, most importantly, the heterogeneity of MND patients (36–38). The same limitations apply to the few longitudinal studies assessing the evolution of cervical cord DTI alterations (27, 36) that showed diverging results about the entity of cord FA decrease over time. One recent study was performed in ALS patients carrying *C9orf72* mutation, demonstrating the spreading of diffusivity alterations from anterior to posterior WM regions over a 6-month period (39).

### Phenotyping the MND Spectrum

DTI measures might also be crucial to distinguish different MND phenotypes. Indeed, DTI metrics were widely used for the identification of “signatures” in PLS. In particular, one study demonstrated that PLS patients showed lower CST FA values relative to healthy controls and ALS patients (40). Degeneration in extra-motor areas has also been found to be similar (41) or even more severe (40) in PLS patients compared to ALS patients. Furthermore, widespread DTI alterations were found to correlate with the severity of cognitive deficits in PLS patients (42). On the other hand, the least extensive microstructural changes were observed in patients with predominant LMN involvement, with diverging results in literature concerning the extent and significance of such damage (43–45). Particularly, a recent two-center study suggested that WM integrity was disrupted along the CST and in frontal and prefrontal regions in patients with predominant LMN disease relative to healthy controls (46). Only patients with predominant LMN involvement and a higher rate of disease progression showed significant WM alterations in the specific ALS-related tract systems (46).

### Clinical and Neuropsychological Correlations

Many DTI studies aimed to test the relationship between WM changes and clinical and neuropsychological measures in MND. Decreased FA in the CST related with disease severity and rate of disease progression in ALS, identifying an association between worsening disability and degeneration of WM tracts, both in the brain (21) and the cervical cord (24, 27). These findings support the potential use of connectivity measures as markers of disease progression in ALS. Inconsistencies among different studies have been reported as for the relationship between DTI measures and disease duration in ALS patients, as longer disease duration has been paradoxically associated with both increased FA (47) and increased MD values of the CST (48). These discrepancies may be explained by the different progression rates of the two samples. DTI changes in the CST and corpus callosum, as well as in the cingulum, inferior longitudinal, inferior fronto-occipital, and uncinate fasciculi have been found to correlate with performance at cognitive tests assessing attention and executive functions (49). Additional extensive WM damage to extra-motor frontotemporal tracts has also been shown, underlying variable degree of behavioral and cognitive disturbances in ALS patients (45, 50, 51). Particularly, one study demonstrated that WM abnormalities of the corpus callosum and frontotemporal tracts, including uncinate, cingulum, and superior longitudinal fasciculi, are the best predictor of executive and non-executive deficits and behavioral changes within the MND spectrum (51).

### Network-Based Analyses

Network-based analysis of structural connections is a new powerful technique that allows studying the brain of healthy subjects or patients with neurodegenerative disorders. The techniques mentioned so far allow to map WM tracts individually using DTI. Recently, neuroimaging research has moved to the study of the human connectome, which aims to map all the possible pathways of the human brain (52). With such new approach, it is possible to provide information about how networks are embedded and interact in the brain. Using graph analysis and connectomics, brain regions can be depicted as a set of nodes, linked by edges representing structural connections. Maps of structural connectivity are created following the following steps: (1) network nodes are identified applying a selected atlas of GM structures to the brain; (2) following definition of the brain regions, WM tracts are reconstructed using DTI; (3) streamlines of the whole brain touching each couple *i* and *j* of the segmented GM nodes are selected; (4) the number of streamlines is calculated for each tract and inserted into a matrix; (5) for each structural connection, the level of microstructural integrity is measured extracting the mean FA, MD, RD, and AD values; (6) finally, all the values are inserted into four different matrices. From the analysis of these matrices, it is possible to provide information concerning the topological organization of network architecture (53). Many studies have examined the global and local graph metrics such as: (1) nodal strength and degree, which provide information regarding the effect of a node in the network; (2) clustering coefficient and local efficiency, which reflect the level of local organization of a network; (3) path length, that is the number of steps needed to connect each pair of nodes; (4) global efficiency, calculated as the inverse of path length, which represents the efficacy of a network to communicate between each pair of nodes; (5) modularity, which gives information regarding segregation of a network, reflecting the level of modular organization (54, 55). To date, modifications of brain topological organization and disruption of structural connectivity have been associated with several neurodegenerative disorders (56–58), including MND.

In a first cross-sectional study, structural brain networks were compared between ALS patients and healthy controls applying network-based statistics (59). ALS patients showed regions with reduced WM connectivity, centered around the primary but also included secondary motor regions (frontal cortex and pallidum). In addition, overall efficiency and clustering coefficient were found to be decreased in ALS patients. A second study studied WM alterations using network analysis, comparing results with those obtained using TBSS (60). The results, consistent with the previous study, showed an impaired motor-frontal-subcortical subnetwork in the ALS patients compared with controls (60). The study also revealed that the results obtained with the network analysis have a strong correspondence with voxel-based approaches (60).

To date, only a few longitudinal studies aimed to investigate the effect of ALS on the brain network over-time. Particularly, one study showed an expanding sub-network of impaired brain connections after six months, with a central role of the primary motor regions (61). The loss of structural connectivity was found to propagate to frontal and parietal regions, supporting the idea that disease spreads along WM connections following a pattern classified into sequential stages (62).

### DTI as a Non-invasive *in-vivo* Biomarker of Disease Spreading

**Figure 1:**
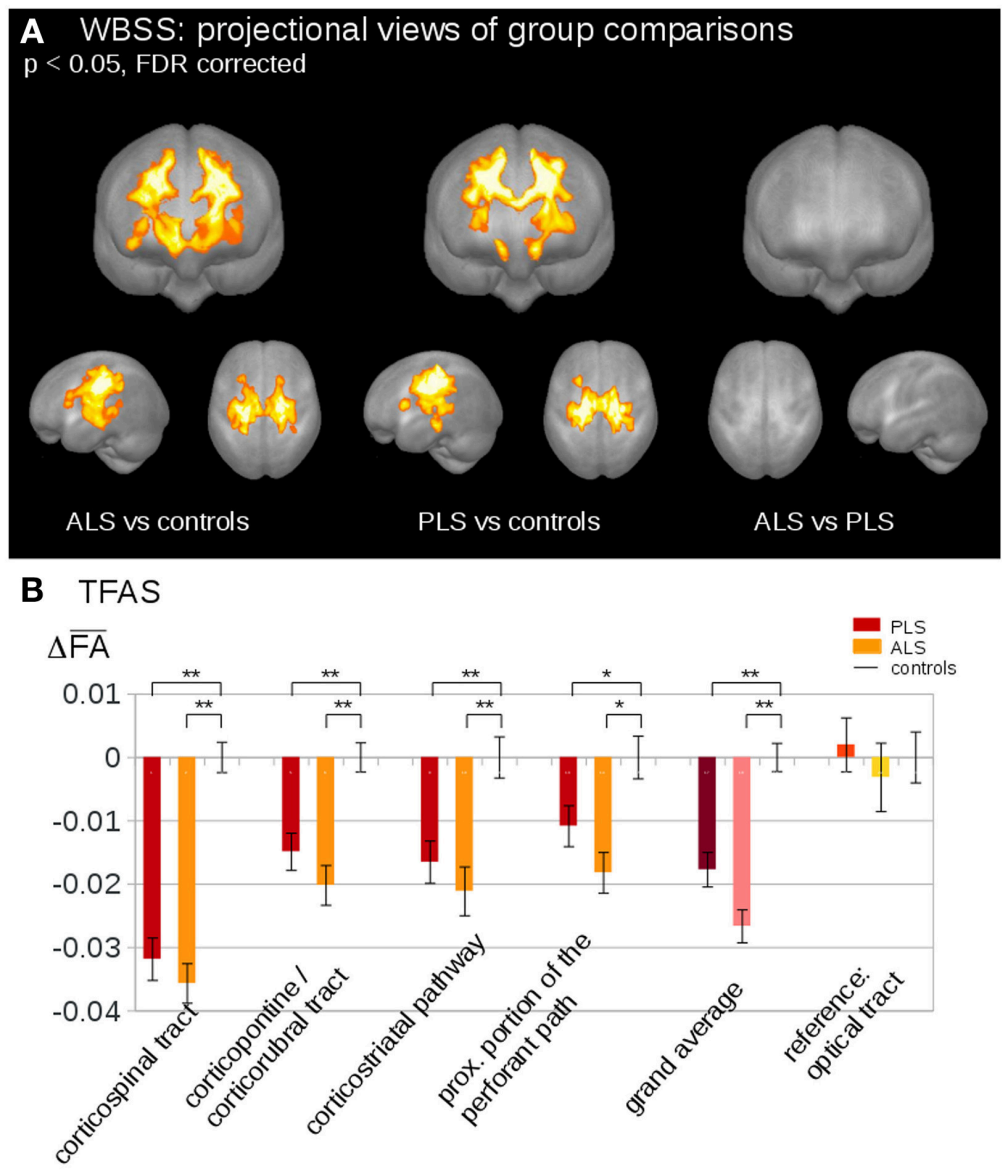
**(A)** Whole brain-based spatial statistics (WBSS) of fractional anisotropy (FA) maps at the group level for amyotrophic lateral sclerosis (ALS) patients, primary lateral sclerosis (PLS) patients, and controls. WBSS of FA maps demonstrated multiple clusters of regional FA reductions at *p* < 0.05 (corrected for multiple comparisons), projectional views. **(B)** Tractwise fractional anisotropy statistics (TFAS) of FA maps at the group level for ALS patients, PLS patients, and controls. TFAS demonstrated significant regional FA reductions in ALS-related tract systems and in the grand average between ALS patients and controls as well as between PLS patients and controls. No alterations between groups were observed in the reference tract. ^*^p < 0.05, ^**^p < 0.001. Reproduced with permission from Müller et al. NeuroImage Clinical 2018 (41) (published open-access under a CC BY-NC-ND 4.0 license).

Neuropathological studies identified the cytoplasmic inclusions of TDP-43 as the molecular hallmark in up to 98% of ALS cases (63). In the last few years, several studies have speculated that the progressive regional accumulation of TDP-43 aggregates in the brain might be reflected by the consecutive deterioration of WM fiber tracts (61). In light of this, DTI-based approaches have been used to study propagation patterns in the brain of MND patients. A DTI study, using a tract of interest-based staging approach, confirmed the neuropathological progression of ALS in the following order: CST (stage 1); corticorubral and corticopontine tracts (stage 2); corticostriatal pathway (stage 3) and proximal portion of the perforant path (stage 4) (64). Furthermore, the extracted tracts of interest were used to categorize ALS patients into the predefined stages according with their WM damage. Staging categorization at baseline was able to classify 72% of the ALS patients into the different stages. After 6 months, there was an increase in ALS stage in 27% of ALS patients (64). Recent studies applied the *in-vivo* staging approach also to phenotypic variants of ALS. One study aimed to figure out if PLS might be a separate disease or just a slowly progressive variant of ALS (41). Microstructural changes were analyzed using the same approach as “classical” ALS, demonstrating that ALS and PLS patients showed identical alterations in the ALS-related tract systems, considering consequently PLS as phenotypical variant of ALS (41) (Figure 1).

**Figure 2:**
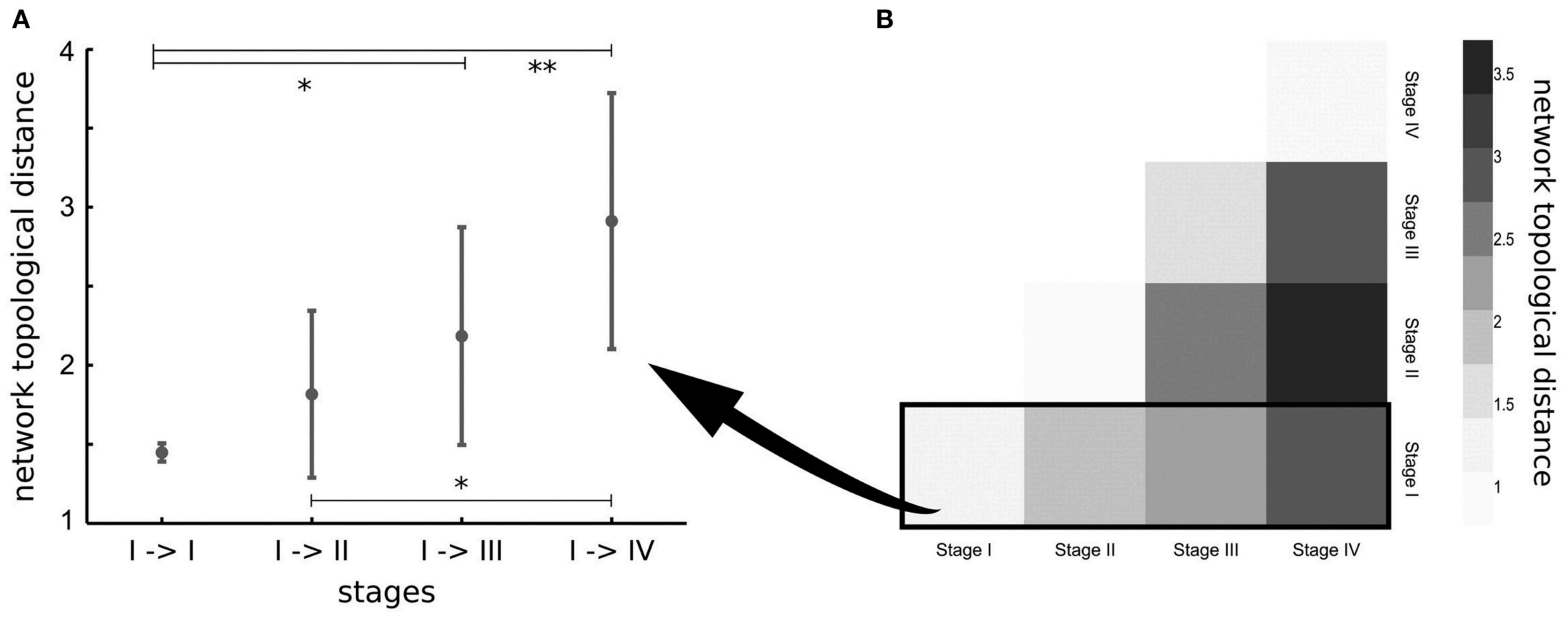
**(A)** Network topological distance between nodes of stage I, stage I and stage II, stages I, and III and between stages I and IV show a strong ordering effect (*p* = 0.002). Significance of differences in network topological distances between stages is marked as follows: ^*^p < 0.05, ^**^p < 0.005. **(B)** Matrix of mean network topological distances between all four stages. Reproduced with permission from Schmidt et al. NeuroImage 2016 (65) (published open-access under a CC BY-NC-ND 4.0 license).

The previously considered studies investigated pathology spreading in ALS-related tracts that were selected *a priori*, according with *post-mortem* neuropathological stages. In order to overcome this *a-priori* selection, one study applied network analysis to investigate the underlying pathogenic mechanism of ALS (65). The results showed that regions involved by TDP-43 pathology in early disease stages are highly structurally interconnected in the brain (65). Furthermore, brain regions of subsequent neuropathological stages were found more closely interconnected than regions of more distant stages (65), suggesting that spread of TDP-43 in ALS occurs along axonal pathways (Figure 2). The DTI-based *in-vivo* staging of MND patients needs to be confirmed in future longitudinal studies with *post-mortem* confirmation.

## Discussion and Future Directions

In the context of therapeutic trials, it is essential to identify a useful biomarker that might help for diagnosis, stratification and tracking the disease progression within the MND spectrum. In order to provide new drugs that could aid the early treatment of the disease, the identification of such biomarker is a crucial point to be addressed. Within such a framework, MRI has been long recognized as *in-vivo* biomarker and, in the last few years, an increasing number of studies applied advanced neuroimaging techniques in order to understand the underlying mechanisms in MND. Particularly, we highlighted the important role of DTI, as a very useful tool in order to characterize microstructural changes during the progression of the disease, to find “signatures” of the different phenotype of MND and to track *in vivo* the progressive spread of TDP-43 aggregates. In order to detect WM changes of different phenotypes of MND, cross-sectional studies were performed highlighting alterations within specific tracts, especially in the CST as well as in the corpus callosum. In light of the fact that decreased FA and increased MD describe the microstructural damage in MND patients, we support the idea that the most potential promising DTI biomarkers are FA or MD changes in the CST and corpus callosum. Additionally, connectivity measures might potentially be considered as a marker of disease progression. This is because decreased FA and disease severity and rate of disease progression are highly correlated. In the last few years, the focus has shifted towards the analysis of disease progression. Particularly, several longitudinal neuroimaging studies are confirming the recently proposed neuropathological staging model (62), demonstrating an expanding subnetwork of impaired brain connections from the primary motor cortex to frontal and parietal regions. All these findings support the idea that WM tract involvement might be a valid biomarker to assess *in vivo* the spreading of pathological proteins and to track the neurodegeneration process.

In conclusion, DTI analysis has the potential to be a valid technique for use at the individual patient level in the future. However, there is urgent need for more longitudinal studies. The combination of the *in vivo* staging using longitudinal DTI scans with the *post-mortem* classification might be very useful to understand deeply the pathophysiology of the disease and to provide as soon as possible disease-modifying therapies.

## Author Contributions

SB drafted the first version of the manuscript. All the authors discussed/edited the draft producing the final version of the manuscript.

### Conflict of Interest Statement

MF is Editor-in-Chief of the Journal of Neurology; received compensation for consulting services and/or speaking activities from Biogen Idec, Merck-Serono, Novartis, Teva Pharmaceutical Industries; and receives research support from Biogen Idec, Merck-Serono, Novartis, Teva Pharmaceutical Industries, Roche, Italian Ministry of Health, Fondazione Italiana Sclerosi Multipla, and ARiSLA (Fondazione Italiana di Ricerca per la SLA). FA is Section Editor of NeuroImage: Clinical; has received speaker honoraria from Biogen Idec and Novartis; and receives or has received research supports from the Italian Ministry of Health, AriSLA (Fondazione Italiana di Ricerca per la SLA), and the European Research Council. The remaining authors declare that the research was conducted in the absence of any commercial or financial relationships that could be construed as a potential conflict of interest.

## References

[1] Rafalowska J Dziewulska D. White matter injury in amyotrophic lateral sclerosis (ALS). Folia Neuropathol. (1996) 34:87–91. 8791897

[2] Smith MC. Nerve fibre degeneration in the brain in amyotrophic lateral sclerosis. J Neurol Neurosurg Psychiatry. (1960) 23:269–82. 10.1136/jnnp.23.4.269 21610893 PMC497425

[3] Le Bihan D Breton E Lallemand D Grenier P Cabanis E Laval-Jeantet M MR imaging of intravoxel incoherent motions: application to diffusion and perfusion in neurologic disorders. Radiology. (1986) 161:401–7. 10.1148/radiology.161.2.3763909 3763909

[4] Basser PJ Mattiello J LeBihan D. Estimation of the effective self-diffusion tensor from the NMR spin echo. J Magn Reson B. (1994) 103:247–54. 10.1006/jmrb.1994.1037 8019776

[5] Snook L Plewes C Beaulieu C. Voxel based versus region of interest analysis in diffusion tensor imaging of neurodevelopment. Neuroimage. (2007) 34:243–52. 10.1016/j.neuroimage.2006.07.021 17070704

[6] Bookstein FL “Voxel-based morphometry” should not be used with imperfectly registered images. Neuroimage. (2001) 14:1454–62. 10.1006/nimg.2001.0770 11707101

[7] Smith SM Jenkinson M Johansen-Berg H Rueckert D Nichols TE Mackay CE. Tract-based spatial statistics: voxelwise analysis of multi-subject diffusion data. Neuroimage. (2006) 31:1487–505. 10.1016/j.neuroimage.2006.02.024 16624579

[8] Iturria-Medina Y Canales-Rodriguez EJ Melie-Garcia L Valdes-Hernandez PA Martinez-Montes E Aleman-Gomez Y. Characterizing brain anatomical connections using diffusion weighted MRI and graph theory. Neuroimage. (2007) 36:645–60. 10.1016/j.neuroimage.2007.02.012 17466539

[9] Douaud G Jbabdi S Behrens TE Menke RA Gass A Monsch AU. DTI measures in crossing-fibre areas: increased diffusion anisotropy reveals early white matter alteration in MCI and mild Alzheimer's disease. Neuroimage. (2011) 55:880–90. 10.1016/j.neuroimage.2010.12.008 21182970 PMC7116583

[10] Barritt AW Gabel MC Cercignani M Leigh PN. Emerging magnetic resonance imaging techniques and analysis methods in amyotrophic lateral sclerosis. Front Neurol. (2018) 9:1065. 10.3389/fneur.2018.01065 30564192 PMC6288229

[11] Trojsi F Caiazzo G Di Nardo F Fratello M Santangelo G Siciliano M. High angular resolution diffusion imaging abnormalities in the early stages of amyotrophic lateral sclerosis. J Neurol Sci. (2017) 380:215–22. 10.1016/j.jns.2017.07.039 28870572

[12] Broad RJ Gabel MC Dowell NG Schwartzman DJ Seth AK Zhang H. Neurite orientation and dispersion density imaging (NODDI) detects cortical and corticospinal tract degeneration in ALS. J Neurol Neurosurg Psychiatry. (2018). [Epub ahead of print]. 10.1136/jnnp-2018-318830 30361295 PMC6581155

[13] Iwata NK Aoki S Okabe S Arai N Terao Y Kwak S. Evaluation of corticospinal tracts in ALS with diffusion tensor MRI and brainstem stimulation. Neurology. (2008) 70:528–32. 10.1212/01.wnl.0000299186.72374.19 18268244

[14] Yin H Cheng SH Zhang J Ma L Gao Y Li D. Corticospinal tract degeneration in amyotrophic lateral sclerosis: a diffusion tensor imaging and fibre tractography study. Ann Acad Med Singapore. (2008) 37:411–5. 18536829

[15] Metwalli NS Benatar M Nair G Usher S Hu X Carew JD. Utility of axial and radial diffusivity from diffusion tensor MRI as markers of neurodegeneration in amyotrophic lateral sclerosis. Brain Res. (2010) 1348:156–64. 10.1016/j.brainres.2010.05.067 20513367

[16] Stagg CJ Knight S Talbot K Jenkinson M Maudsley AA Turner MR. Whole-brain magnetic resonance spectroscopic imaging measures are related to disability in ALS. Neurology. (2013) 80:610–5. 10.1212/WNL.0b013e318281ccec 23325907 PMC3590062

[17] Sarica A Cerasa A Vasta R Perrotta P Valentino P Mangone G. Tractography in amyotrophic lateral sclerosis using a novel probabilistic tool: a study with tract-based reconstruction compared to voxel-based approach. J Neurosci Methods. (2014) 224:79–87. 10.1016/j.jneumeth.2013.12.014 24406465

[18] Müller HP Turner MR Grosskreutz J Abrahams S Bede P Govind V. A large-scale multicentre cerebral diffusion tensor imaging study in amyotrophic lateral sclerosis. J Neurol Neurosurg Psychiatry. (2016) 87:570–9. 10.1136/jnnp-2015-311952 26746186

[19] Thivard L Pradat PF Lehericy S Lacomblez L Dormont D Chiras J. Diffusion tensor imaging and voxel based morphometry study in amyotrophic lateral sclerosis: relationships with motor disability. J Neurol Neurosurg Psychiatry. (2007) 78:889–92. 10.1136/jnnp.2006.101758 17635981 PMC2117724

[20] Canu E Agosta F Riva N Sala S Prelle A Caputo D. The topography of brain microstructural damage in amyotrophic lateral sclerosis assessed using diffusion tensor MR imaging. AJNR Am J Neuroradiol. (2011) 32:1307–14. 10.3174/ajnr.A2469 21680655 PMC7966037

[21] Keil C Prell T Peschel T Hartung V Dengler R Grosskreutz J. Longitudinal diffusion tensor imaging in amyotrophic lateral sclerosis. BMC Neurosci. (2012) 13:141. 10.1186/1471-2202-13-141 23134591 PMC3531302

[22] Sach M Winkler G Glauche V Liepert J Heimbach B Koch MA. Diffusion tensor MRI of early upper motor neuron involvement in amyotrophic lateral sclerosis. Brain. (2004) 127:340–50. 10.1093/brain/awh041 14607785

[23] Agosta F Pagani E Rocca MA Caputo D Perini M Salvi F. Voxel-based morphometry study of brain volumetry and diffusivity in amyotrophic lateral sclerosis patients with mild disability. Hum Brain Mapp. (2007) 28:1430–8. 10.1002/hbm.20364 17370339 PMC6871473

[24] Valsasina P Agosta F Benedetti B Caputo D Perini M Salvi F. Diffusion anisotropy of the cervical cord is strictly associated with disability in amyotrophic lateral sclerosis. J Neurol Neurosurg Psychiatry. (2007) 78:480–4. 10.1136/jnnp.2006.100032 17030586 PMC2117814

[25] Nair G Carew JD Usher S Lu D Hu XP Benatar M. Diffusion tensor imaging reveals regional differences in the cervical spinal cord in amyotrophic lateral sclerosis. Neuroimage. (2010) 53:576–83. 10.1016/j.neuroimage.2010.06.060 20600964

[26] Cohen-Adad J Zhao W Keil B Ratai EM Triantafyllou C Lawson R. 7-T MRI of the spinal cord can detect lateral corticospinal tract abnormality in amyotrophic lateral sclerosis. Muscle Nerve. (2013) 47:760–2. 10.1002/mus.23720 23553571

[27] El Mendili MM Cohen-Adad J Pelegrini-Issac M Rossignol S Morizot-Koutlidis R Marchand-Pauvert V Multi-parametric spinal cord MRI as potential progression marker in amyotrophic lateral sclerosis. PLoS ONE. (2014) 9:e95516 10.1371/journal.pone.0095516 24755826 PMC3995720

[28] Bede P Bokde AL Byrne S Elamin M McLaughlin RL Kenna K. Multiparametric MRI study of ALS stratified for the C9orf72 genotype. Neurology. (2013) 81:361–9. 10.1212/WNL.0b013e31829c5eee 23771489 PMC3772833

[29] Agosta F Ferraro PM Riva N Spinelli EG Domi T Carrera P. Structural and functional brain signatures of C9orf72 in motor neuron disease. Neurobiol Aging. (2017) 57:206–19. 10.1016/j.neurobiolaging.2017.05.024 28666709

[30] Stanton BR Shinhmar D Turner MR Williams VC Williams SC Blain CR. Diffusion tensor imaging in sporadic and familial (D90A SOD1) forms of amyotrophic lateral sclerosis. Arch Neurol. (2009) 66:109–15. 10.1001/archneurol.2008.527 19139308

[31] Agosta F Spinelli EG Marjanovic IV Stevic Z Pagani E Valsasina P. Unraveling ALS due to SOD1 mutation through the combination of brain and cervical cord MRI. Neurology. (2018) 90:e707–16. 10.1212/WNL.0000000000005002 29367447

[32] van der Graaff MM Sage CA Caan MW Akkerman EM Lavini C Majoie CB Upper and extra-motoneuron involvement in early motoneuron disease: a diffusion tensor imaging study. Brain. (2011) 134:1211–28. 10.1093/brain/awr016 21362631

[33] Menke RA Korner S Filippini N Douaud G Knight S Talbot K. Widespread grey matter pathology dominates the longitudinal cerebral MRI and clinical landscape of amyotrophic lateral sclerosis. Brain. (2014) 137:2546–55. 10.1093/brain/awu162 24951638 PMC4132644

[34] Bede P Elamin M Byrne S McLaughlin RL Kenna K Vajda A. Patterns of cerebral and cerebellar white matter degeneration in ALS. J Neurol Neurosurg Psychiatry. (2015) 86:468–70. 10.1136/jnnp-2014-308172 25053771 PMC4392231

[35] Cardenas-Blanco A Machts J Acosta-Cabronero J Kaufmann J Abdulla S Kollewe K. Structural and diffusion imaging versus clinical assessment to monitor amyotrophic lateral sclerosis. Neuroimage Clin. (2016) 11:408–14. 10.1016/j.nicl.2016.03.011 27104135 PMC4827722

[36] Agosta F Rocca MA Valsasina P Sala S Caputo D Perini M. A longitudinal diffusion tensor MRI study of the cervical cord and brain in amyotrophic lateral sclerosis patients. J Neurol Neurosurg Psychiatry. (2009) 80:53–5. 10.1136/jnnp.2008.154252 18931009

[37] Kwan JY Meoded A Danielian LE Wu T Floeter MK. Structural imaging differences and longitudinal changes in primary lateral sclerosis and amyotrophic lateral sclerosis. Neuroimage Clin. (2012) 2:151–60. 10.1016/j.nicl.2012.12.003 24179768 PMC3778247

[38] Menke RA Agosta F Grosskreutz J Filippi M Turner MR. Neuroimaging endpoints in amyotrophic lateral sclerosis. Neurotherapeutics. (2017) 14:11–23. 10.1007/s13311-016-0484-9 27752938 PMC5233627

[39] Floeter MK Danielian LE Braun LE Wu T. Longitudinal diffusion imaging across the C9orf72 clinical spectrum. J Neurol Neurosurg Psychiatry. (2018) 89:53–60. 10.1136/jnnp-2017-316799 29054917 PMC6454927

[40] Agosta F Galantucci S Riva N Chio A Messina S Iannaccone S. Intrahemispheric and interhemispheric structural network abnormalities in PLS and ALS. Hum Brain Mapp. (2014) 35:1710–22. 10.1002/hbm.22286 23633431 PMC6869498

[41] Müller HP Agosta F Gorges M Kassubek R Spinelli EG Riva N. Cortico-efferent tract involvement in primary lateral sclerosis and amyotrophic lateral sclerosis: a two-centre tract of interest-based DTI analysis. Neuroimage Clin. (2018) 20:1062–9. 10.1016/j.nicl.2018.10.005 30343251 PMC6198122

[42] Canu E Agosta F Galantucci S Chio A Riva N Silani V. Extramotor damage is associated with cognition in primary lateral sclerosis patients. PLoS ONE. (2013) 8:e82017. 10.1371/journal.pone.0082017 24349172 PMC3857796

[43] Prudlo J Bissbort C Glass A Grossmann A Hauenstein K Benecke R. White matter pathology in ALS and lower motor neuron ALS variants: a diffusion tensor imaging study using tract-based spatial statistics. J Neurol. (2012) 259:1848–59. 10.1007/s00415-012-6420-y 22349938

[44] Rosenbohm A Müller HP Hubers A Ludolph AC Kassubek J. Corticoefferent pathways in pure lower motor neuron disease: a diffusion tensor imaging study. J Neurol. (2016) 263:2430–7. 10.1007/s00415-016-8281-2 27624123

[45] Spinelli EG Agosta F Ferraro PM Riva N Lunetta C Falzone YM. Brain MR imaging in patients with lower motor neuron-predominant disease. Radiology. (2016) 280:545–56. 10.1148/radiol.2016151846 26963576

[46] Müller HP Agosta F Riva N Spinelli EG Comi G Ludolph AC. Fast progressive lower motor neuron disease is an ALS variant: a two-centre tract of interest-based MRI data analysis. Neuroimage Clin. (2018) 17:145–52. 10.1016/j.nicl.2017.10.008 29071208 PMC5651542

[47] Iwata NK Kwan JY Danielian LE Butman JA Tovar-Moll F Bayat E. White matter alterations differ in primary lateral sclerosis and amyotrophic lateral sclerosis. Brain. (2011) 134:2642–55. 10.1093/brain/awr178 21798965 PMC3170531

[48] Ellis CM Simmons A Jones DK Bland J Dawson JM Horsfield MA. Diffusion tensor MRI assesses corticospinal tract damage in ALS. Neurology. (1999) 53:1051–8. 10.1212/WNL.53.5.1051 10496265

[49] Sarro L Agosta F Canu E Riva N Prelle A Copetti M. Cognitive functions and white matter tract damage in amyotrophic lateral sclerosis: a diffusion tensor tractography study. AJNR Am J Neuroradiol. (2011) 32:1866–72. 10.3174/ajnr.A2658 22016410 PMC7966026

[50] Lillo P Mioshi E Burrell JR Kiernan MC Hodges JR Hornberger M. Grey and white matter changes across the amyotrophic lateral sclerosis-frontotemporal dementia continuum. PLoS ONE. (2012) 7:e43993. 10.1371/journal.pone.0043993 22952843 PMC3430626

[51] Agosta F Ferraro PM Riva N Spinelli EG Chio A Canu E. Structural brain correlates of cognitive and behavioral impairment in MND. Hum Brain Mapp. (2016) 37:1614–26. 10.1002/hbm.23124 26833930 PMC6867462

[52] Bullmore E Sporns O. Complex brain networks: graph theoretical analysis of structural and functional systems. Nat Rev Neurosci. (2009) 10:186–98. 10.1038/nrn2575 19190637

[53] Stam CJ van Straaten EC. The organization of physiological brain networks. Clin Neurophysiol. (2012) 123:1067–87. 10.1016/j.clinph.2012.01.011 22356937

[54] Watts DJ Strogatz SH. Collective dynamics of ‘small-world’ networks. Nature. (1998) 393:440–2. 10.1038/30918 9623998

[55] Sporns O Chialvo DR Kaiser M Hilgetag CC. Organization, development and function of complex brain networks. Trends Cogn Sci. (2004) 8:418–25. 10.1016/j.tics.2004.07.008 15350243

[56] Filippi M Basaia S Canu E Imperiale F Meani A Caso F. Brain network connectivity differs in early-onset neurodegenerative dementia. Neurology. (2017) 89:1764–72. 10.1212/WNL.0000000000004577 28954876 PMC5664301

[57] Galantucci S Agosta F Stefanova E Basaia S van den Heuvel MP Stojkovic T. Structural brain connectome and cognitive impairment in parkinson disease. Radiology. (2017) 283:515–25. 10.1148/radiol.2016160274 27924721

[58] Filippi M Basaia S Canu E Imperiale F Magnani G Falautano M. Changes in functional and structural brain connectome along the Alzheimer's disease continuum. Mol Psychiatry. (2018). [Epub ahead of print]. 10.1038/s41380-018-0067-8 29743583

[59] Verstraete E Veldink JH Mandl RC van den Berg LH van den Heuvel MP. Impaired structural motor connectome in amyotrophic lateral sclerosis. PLoS ONE. (2011) 6:e24239. 10.1371/journal.pone.0024239 21912680 PMC3166305

[60] Buchanan CR Pettit LD Storkey AJ Abrahams S Bastin ME. Reduced structural connectivity within a prefrontal-motor-subcortical network in amyotrophic lateral sclerosis. J Magn Reson Imaging. (2015) 41:1342–52. 10.1002/jmri.24695 25044733

[61] Verstraete E Veldink JH van den Berg LH van den Heuvel MP. Structural brain network imaging shows expanding disconnection of the motor system in amyotrophic lateral sclerosis. Hum Brain Mapp. (2014) 35:1351–61. 10.1002/hbm.22258 23450820 PMC6869230

[62] Brettschneider J Del Tredici K Toledo JB Robinson JL Irwin DJ Grossman M. Stages of pTDP-43 pathology in amyotrophic lateral sclerosis. Ann Neurol. (2013) 74:20–38. 10.1002/ana.23937 23686809 PMC3785076

[63] Neumann M Sampathu DM Kwong LK Truax AC Micsenyi MC Chou TT. Ubiquitinated TDP-43 in frontotemporal lobar degeneration and amyotrophic lateral sclerosis. Science. (2006) 314:130–3. 10.1126/science.1134108 17023659

[64] Kassubek J Müller HP Del Tredici K Brettschneider J Pinkhardt EH Lule D. Diffusion tensor imaging analysis of sequential spreading of disease in amyotrophic lateral sclerosis confirms patterns of TDP-43 pathology. Brain. (2014) 137:1733–40. 10.1093/brain/awu090 24736303

[65] Schmidt R de Reus MA Scholtens LH van den Berg LH van den Heuvel MP. Simulating disease propagation across white matter connectome reveals anatomical substrate for neuropathology staging in amyotrophic lateral sclerosis. Neuroimage. (2016) 124:762–9. 10.1016/j.neuroimage.2015.04.005 25869856

